# Identifying Shared Key Genes and Cellular Characteristics Between Chronic Periodontitis and Aging Using Integrative Single‐Cell and Mendelian Randomization Analyses

**DOI:** 10.1155/bmri/5156800

**Published:** 2026-06-17

**Authors:** Fengzhen Lei, Rui Shi, Qiuli Chen, Zelong Zeng, Chen Sun, Mei He

**Affiliations:** ^1^ Department of Stomatology, Shenzhen Longhua District Central Hospital, Shenzhen, China, glyy.org; ^2^ Department of Periodontics and Oral Mucosal Diseases, The Affiliated Stomatology Hospital of Southwest Medical University, Luzhou, Sichuan, China

**Keywords:** aging, chronic periodontitis, MR, single-cell

## Abstract

**Objective:**

The purpose was to investigate the shared key genes between chronic periodontitis (CP) and aging.

**Materials and Methods:**

First, we collected single‐cell RNA sequencing (scRNA‐seq) data of peripheral blood samples from patients with CP, aging, and healthy control (HC) and confirmed the cross‐group coexpressed and downregulated cell subsets as the core research objects. Subsequently, we explored the dynamic evolutionary rules and functional interaction patterns of these cell subsets by integrating pseudotime trajectory analysis and intercellular communication modeling. Then, we screened the differentially expressed gene targets and adopted mendelian randomization (MR) analysis to clarify causal association between aging and CP susceptibility. Finally, we performed colocalization analysis by integrating data from CP genome‐wide association studies (GWAS) and expression quantitative trait locus datasets and conducted metabolic pathway enrichment analysis on the key genes obtained.

**Results:**

Subsets of T cells were found to account for a significant proportion in both CP patients and aging individuals. CD8_CM cells showed a downward trend in these two groups, screening of 16 differentially expressed genes. MR indicated that *GZMK, KLRG1,* and *LYAR* had causal relationships and were retained in the validation phase, and colocalization analysis yielded limited evidence supporting the existence of shared causal variants. Furthermore, metabolic enrichment analysis delineated the metabolic pathways functionally associated with these genes.

**Conclusion:**

This study found the downregulation of CD8_CM cells is a shared feature of CP and aging, and pointed out that *GZMK, KLRG1* and *LYAR* may be the molecular links, offering an innovative perspective on elucidating the intersection mechanisms underlying aging and CP.

## 1. Introduction

Chronic periodontitis (CP) is a prevalent inflammatory disease characterized by progressive destruction of periodontal tissues, with aging recognized as a key risk factor that accelerates disease progression. The interplay between CP and aging is bidirectional. Although aging exacerbates periodontal inflammation and alveolar bone loss, CP‐induced chronic inflammation contributes to systemic aging processes, particularly through inflammaging, a state of low‐grade sterile inflammation associated with aging [1–4]. Despite increasing recognition of this relationship, the molecular mechanisms linking CP and aging remain incompletely understood, underscoring the need for integrative approaches to dissect shared pathways and cellular dynamics [5].

Aging disrupts immune homeostasis and tissue repair, rendering periodontal tissues more vulnerable to microbial insults. Cellular senescence has emerged as a critical mediator in this process. There is a gradual accumulation of aging osteocytes with advancing age, which elaborate pro‐inflammatory cytokines such as tumor necrosis factor‐*α* (TNF‐*α*) and interleukin‐6 (IL‐6) via engagement of the senescence‐associated secretory phenotype (SASP) [1]. SASP‐driven inflammation not only exacerbates local tissue destruction but also induces secondary senescence in neighboring cells, creating a self‐perpetuating cycle [1]. Concurrently, impaired immune clearance of senescent cells and mitochondrial dysfunction further amplify tissue damage [6, 7]. Recent studies also demonstrate that aging gingival tissues exhibit elevated hypoxia‐inducible factors (HIF‐1*α*/HIF‐2*α*), which dysregulate metabolic pathways and reinforce pro‐inflammatory phenotypes, highlighting aging as a multifaceted driver of periodontal pathology [3].

Inflammaging represents a converging pathway between CP and aging. This hallmark of aging is characterized by chronic activation of innate immune pathways (e.g., NF‐*κ*B, NLRP3 inflammasome) and persistent elevation of cytokines such as IL‐1*β* and IL‐6 [8]. In CP, periodontal pathogens (e.g., *Porphyromonas gingivalis*) and their virulence factors such as lipopolysaccharide (LPS) exacerbate inflammaging by synergizing with age‐related immune dysregulation [9]. For example, TLR4‐mediated NLRP3 activation in gingival fibroblasts suppresses BMI‐1, a regulator of cellular senescence, thereby promoting SASP and fueling chronic inflammation [8]. Systemically, CP‐derived inflammatory mediators (e.g., C‐reactive protein) disseminate into circulation, accelerating age‐related comorbidities such as cardiovascular disease and metabolic syndrome [10]. This bidirectional crosstalk positions inflammaging as a central hub connecting local periodontal pathology with systemic aging.

Traditional bulk RNA sequencing lacks the resolution to capture cellular heterogeneity in periodontal tissues, where diverse subsets (e.g., osteoblasts, immune cells) exhibit distinct responses to aging and microbial challenge [11]. The single‐cell RNA sequencing (scRNA‐seq) circumvents this constraint by profiling transcriptomes at the single‐cell resolution, thereby facilitating the identification of rare cell populations and characterization of dynamic molecular states. Recent applications in CP have revealed senescence‐associated transcriptional changes in osteocytes and fibroblasts, as well as altered immune cell interactions in inflamed tissues [1]. For instance, scRNA‐seq studies identified senescent keratinocytes in periodontal pockets that secrete CXCL3 and IL‐1*β*, driving neutrophil recruitment and tissue destruction [12]. Integrative bioinformatics further enhances mechanistic insights by combining scRNA‐seq, bulk transcriptomics, and functional enrichment analyses. Overlaying aging‐related gene signatures (e.g., SASP factors, mitochondrial dysfunction markers) onto CP datasets highlights conserved pathways such as mTOR signaling and oxidative stress [13]. This synergy between high‐resolution cellular profiling and systems‐level analysis offers unique opportunities to identify therapeutic targets (e.g., senolytics, immunomodulatory agents) that address both CP and aging [13].

Although inflammation and senescence have been implicated in CP and aging, the precise molecular interactions and cell‐specific drivers remain incompletely defined. For example, how aging modulates the microbiome–immune interface in periodontal tissues, or whether aging cells in CP contribute to systemic inflammaging, are open questions [14]. Mendelian randomization (MR) is a method used in epidemiology to examine the causal impact of exposures on outcomes by using measured genetic variations [15]. Unlike other types of risk indicators that are prone to being confounded by multiple covariates, these genetic predictors possess the salient feature that the associations of the focal genes with disease development are impervious to the influence of commonly encountered confounding variables [16, 17]. Moreover, applications of scRNA‐seq in aging research remain limited, particularly with respect to longitudinal analyses of disease progression [18].

For the purposes of this investigation, we performed an integrative analysis of scRNA‐seq data obtained from a cohort of clinical patients with CP and aging cohorts to uncover common mechanisms underlying their immunopathogenesis. To further dissect the potential causal relationships between key candidate genes and CP, we applied two‐sample MR analysis. Notably, the characterization of these core genes and their enriched pathways could shed new light on the common genetic architecture underlying the pathogenesis of CP and the biological processes of aging.

## 2. Materials and Methods

All datasets utilized for the present analyses were retrieved from publicly available, peer‐reviewed repositories, and thus no further ethical approval or informed authorization was deemed necessary.

### 2.1. Data Source

A public functional genomics data repository adhering to MIAME guidelines, the Gene Expression Omnibus (GEO, https://www.ncbi.nlm.nih.gov/geo/) houses an extensive repertoire of next‐generation sequencing and array‐derived datasets associated with diverse disease entities. In the current study, we retrieved the required gene expression datasets of CP and aging from this repository leveraging its built‐in data query and download functionalities. After performing the keywords “chronic periodontitis” and “aging”, we selected four case samples and two control sample from GSE244515 (GSM7818495, GSM7818496, GSM7818506, GSM7818507, GSM7818508, and GSM7818509) for CP and GSE157007 (GSM4750303, GSM4750304, GSM4750308, GSM4750309, GSM4750310, and GSM4750311) for aging to analyze, respectively. Additionally, the bulk RNA‐seq data (GSE173082) were used to validate findings related to key genes of special subtype cells.

Moreover, for the purpose of complementing gene expression analysis, CP‐related genome‐wide association studies (GWAS) data were obtained and incorporated into the study from the GWAS Catalog (http://www.ebi.ac.uk/gwas). The training dataset for CP consists of 455,398 European participants, including 950 cases (GCST90044102). In addition, a validation set for CP was utilized, comprising 57,201 Hispanic or Latin American participants, including 4184 cases (GCST90478264).

### 2.2. scRNA‐seq Analysis

We consolidated and evaluated scRNA‐seq data isolated from peripheral blood specimens collected from patients with CP, aging, and HC. We conducted a comprehensive scRNA‐seq analysis utilizing the Seurat R package (Version 4.4.0) in the present study, including data preprocessing, quality control, dimensionality reduction, clustering, cell‐type annotation, trajectory inference, and intercellular communication analysis.

Firstly, during data quality, cells harboring below 500 or more than 4000 detected genes, as well as those with mitochondrial gene expression surpassing 10%, were excluded from subsequent analyses, as calculated using the PercentageFeatureSet function. These selection rules efficiently eliminated cells with potential poor quality and those predisposed to apoptosis. Log‐normalization was implemented using the default algorithm integrated into Seurat, which was subsequently followed by the identification of highly variable genes to facilitate downstream analytical procedures. For dimensionality reduction, principal component analysis (PCA) was performed, with the top 10 highest‐ranking principal components retained in preparation for subsequent analyses. This was followed by uniform manifold approximation and projection (UMAP) to characterize the global cellular atlas. Subsequently, to mitigate batch effects among multiple samples, we applied Harmony integration, which improved alignment of shared cell types across datasets. Cell clustering analysis was implemented utilizing the FindNeighbors and FindClusters algorithms integrated in Seurat, with the clustering resolution set to 0.5, which yielded biologically interpretable clusters. Finally, cell type annotation was accomplished through the integration of the SingleR algorithm (Version 2.8.0), supplemented by canonical marker gene expression profiling for enhanced annotation [19].

To analyze T cell subsets, we extracted the T cell population from the scRNA‐seq dataset using Seurat′s subset() function. Subsequently, we performed the same dimensionality reduction steps and cell type annotation by singleR and manual curation based on established canonical marker genes. To further investigate lineage relationships and developmental trajectories, we converted the Seurat object to a SingleCellExperiment object and applied the Slingshot algorithm. The slingshot function was used with UMAP embeddings and pre‐defined starting clusters, enabling us to infer lineage relationships among T cell subsets. To investigate intercellular communication among core subpopulations, we employed the CellChat framework. We constructed a unified dataset by integrating T cells with other cell types, stratified it by tissue type (“CP” or “aging”), and downsampled to 2000 cells for computational efficiency. Using CellChat, we inferred and analyzed cell‐cell communication networks based on known ligand‐receptor interactions, focusing on secreted signaling pathways. Low‐abundance interactions were filtered to ensure statistical robustness. The human CellChatDB was filtered for signaling pathways. We then identified overexpressed genes and ligand‐receptor pairs, projected these onto the human protein–protein interaction (PPI) network, and calculated intercellular communication probabilities. Finally, communications with low cell counts were filtered out to retain statistically robust interactions.

### 2.3. MR and Colocalization Analysis

A dual‐sample MR analysis was performed to explore the putative causal association between candidate genes and CP. We adopted the inverse variance weighted (IVW) protocol as the primary analytical methodology for causal inference. To guarantee the robustness and validity of the instrumental variables (IVs), we implemented a comprehensive and rigorous selection procedure, including the identification of strong and independent eQTLs associated with gene expression.

First, to identify key marker genes without prior assumptions about representative subtypes, we first compared this subtype to other T cell subtypes and non‐T cells. The intersection of differentially expressed genes (DEGs) from both comparisons was considered as key markers for the subtype as exposure. Subsequently, single nucleotide polymorphisms (SNPs) were ascertained that were showing a statistically verifiable correlation with the exposure (*p* < 5 × 10^8^) from GWAS. To counteract the bias arising from weak instruments, we computed the F‐statistic (*F* = *β*2/se2) for each SNP and selected only those with an F‐value exceeding 10, thus filtering out weak instruments and improving the validity of causal inference [20]. The reliability of the candidate SNPs was corroborated through cross‐validation against distinct datasets to confirm the persistence of their associations with the exposure. In addition, a reverse MR analysis was conducted to explore potential mutual causal associations, evaluating the potential influence of CP on the exposure in the reverse direction. To adjust for potential presence of horizontal pleiotropy, sensitivity analyses including MR‐Egger regression, leave‐one‐out analyses, and weighted median analysis were incorporated, all configured to identify and alleviate pleiotropic interference. The implementation of these procedures successfully proved that the IVs fulfilled the essential requirements of MR (relevance, exclusion restriction, and independence), thus elevating the trustworthiness from our experimental observations pertaining to the potential causal link between the candidate genes and CP. Finally, to further corroborate our causal inference, colocalization analysis was conducted via the coloc package (Version 5.2.3) [19], targeting the overlapping susceptibility signals between CP GWAS data and key gene eQTL datasets. The posterior likelihood of causal genetic variants overlap across the two distinct traits under investigation was derived from the H4 test statistic yielded by the coloc.abf function, serving as the key outcome indicator.

### 2.4. Expression Quantitative Trait Locus and Steiger Filtering

Complementing our GWAS findings, we additionally performed regional association mapping to explore the SNP genotype‐phenotype correlations for key genes. To explore gene expression associations, we used the LDlink website as an alternative to Phenoscanner function, leveraging its resources to identify relevant SNP‐gene expression relationships. To assess the causal relationship directionality, we applied Steiger filtering to compare the *R* ^2^ values between SNP‐exposure and SNP‐outcome associations. This approach helped verify the correct causal direction by ensuring that the SNPs closer to the exposure in the causal chain had higher *R* ^2^ values compared to those closer to the outcome.

### 2.5. Trajectory, Cell Communication, Metabolic Activity, and Bulk Analysis

We performed various bioinformatics analyses on scRNA‐seq and bulk RNA‐seq data. Key genes were identified from the data, and expression patterns were visualized using DotPlot and FeaturePlot for relevant genes. Trajectory analysis was conducted using the Slingshot method to study the pseudotime progression of special subgroup of T cells, revealing gene expression dynamics. Gene switches, identified using logistic regression, were analyzed to find genes that exhibit switching behavior during the differentiation process. Cell‐cell communication analysis was carried out using the CellChat package, focusing on interactions involving key gene positive and negative subtypes of T cells. Metabolism‐related pathways were evaluated using the scMetabolism package (Version 0.2.1), and the metabolic activity of macrophages was assessed. Differential gene expression and pathway enrichment analyses were performed to highlight important markers and pathways associated with key gene positive subtype. Finally, to characterize the transcriptomic landscape of CP, bulk RNA‐seq data were processed with differential expression profiling to verify the DEGs between HC and CP samples.

### 2.6. Statistical Analysis

All analyses were performed using R (Version 4.5.0; https://www.r‐project.org/). A two‐tailed *p* value < 0.05 was regarded as statistically significant, and adjustments for multiple comparisons were applied as deemed appropriate.

## 3. Results

## 4. scRNA‐seq Analysis

**Figure 1:**
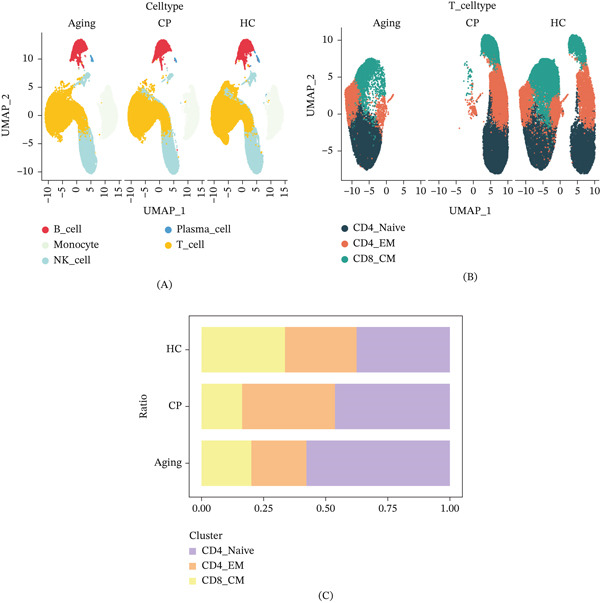
Analysis of scRNA‐seq data for chronic periodontitis (CP), aging and healthy control (HC) groups: (A) demonstrates the proportion of distinct immune cell subsets across each disease group; (B) demonstrates the proportion of distinct T‐cell subsets across each disease group; (C) demonstrates bar charts depicting the ratio of distinct T‐cell subsets among CP, aging, and HC groups. Naive (naive T cells), EM (effector memory T cell), CM (central memory T cell).

**Figure 2:**
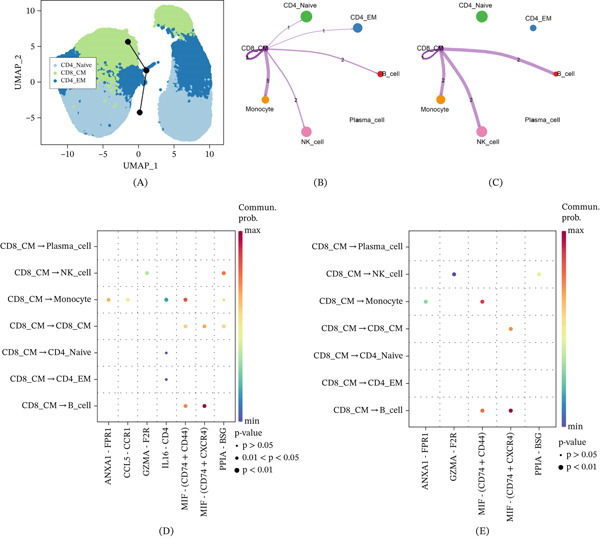
Intercellular communication profiles between CD8_CM cells and other immune cell subsets in chronic periodontitis (CP) versus aging groups: (A) shows cell trajectory analysis; (B) and (C) depict circle plots summarizing the trajectory analysis between CD8_CM and other cell types across the CP and aging groups; (D) and (E) depict the cell‐cell communication network of CD8_CM cells with other subpopulations in the CP and aging groups.

As shown in Figure 1A, T‐cell subsets account for a significant proportion of both patients with CP and aging population. Extensive research has revealed that T‐cell subsets serve as key mediators in the progression of CP and the physiological process of aging. Therefore, T‐cell subsets were taken as the focus of subsequent research. Compared with HC, the CD8_CM cell subset was significantly decreased in patients with CP and aging groups (Figure 1B,C). Figure 2A,B,C displays the trajectory relationships between the CD8_CM and other cell subsets under CP and aging conditions. Systematic cell‐cell communication profiling (Figures 2D,E) revealed that CD8_CM cell subset interacts with other cell subsets and exerts a critical influence on CP and aging diseases. By conducting a comparative analysis of the core cell subset CD8_CM with other T‐cell subsets and non‐T cell types, we screened out 525 and 40 potential marker genes, respectively. Through the intersection of the above two groups, we obtained 16 differential genes.

### 4.1. MR

Subsequent MR analysis of the 16 previously identified DEGs revealed that three of these genes were causally associated with the risk of CP. Figure 3A demonstrates the genes associated with aging in the CD8_CM cell subset that have a causal relationship with CP. Specifically, as depicted in Figure 3B, the IVW method confirmed that the following 3 genes were causally associated with CP: *KLRG1* (nsnp = 1, OR = 5.4781, 95% CI: 1.0316–29.0895, *p* = 0.046), *GZMK*(nsnp = 1, OR = 2.6802, 95% CI: 1.0182–7.0548, *p* = 0.046), *LYAR*(nsnp = 1, OR = 6.2895, 95% CI: 1.0342–38.2491, *p* = 0.046). Validation of the MR analysis results in this study were provided by the results of heterogeneity and horizontal pleiotropy tests. Additionally, no evidence of horizontal pleiotropy was uncovered via the MR‐Egger regression intercept test, as all interceptions yielded nonsignificant statistical results across all analyses, with all corresponding *p* values exceeding 0.05.

Figure 3Mendelian randomization (MR) analysis presents: (A) a forest plot summarizing the MR findings for key genes associated with chronic periodontitis (CP); (B) the MR of key genes with CP; (C) the results of MR of key genes in the validation set with CP; (D) the reverse MR of *GZMK* with CP; (E) the colocalization results of *GZMK*, *KLRG1*, *LYAR*.

**Figure:**
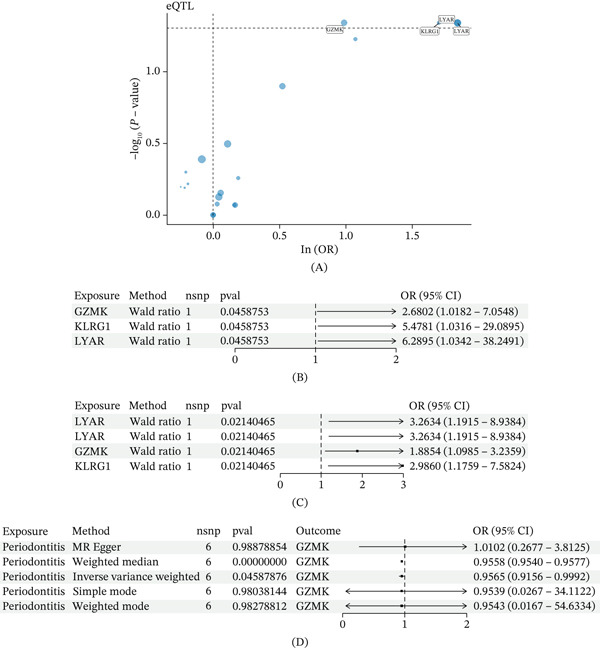
(a)

**Figure:**
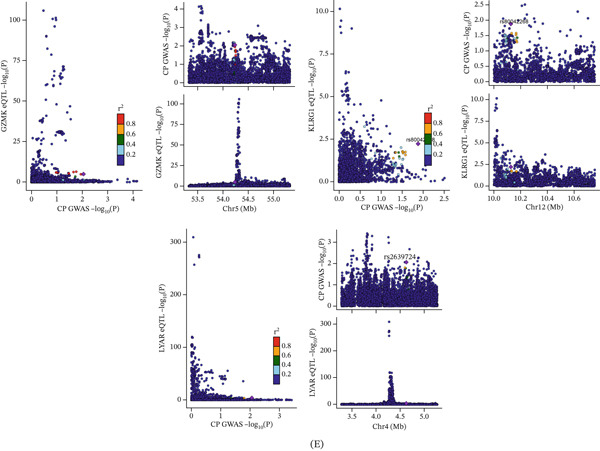
(b)

To confirm the robustness of our findings, we filtered GWAS data from additional CP study as a validation set, and the relevant outcomes are indicated in Figure 3C. Analysis using the IVW method revealed that *KLRG1* (nsnp = 1, OR = 2.9860, 95% CI: 1.1759–7.5824, *p* = 0.021), *GZMK* (nsnp = 1, OR = 1.8854, 95% CI: 1.0985–3.2359, *p* = 0.021) and *LYAR* (nsnp = 1, OR = 3.2634, 95% CI: 1.1915–8.9384, *p* = 0.021) were causally associated with CP. Reverse MR (Figure 3D) showed that *GZMK* (nsnp = 6, OR = 0.9565, 95% CI: 0.9156–0.9992, *p* = 0.046). The results of MR analyses indicated that the *KLRG1, GZMK,* and *LYAR* genes have a causal relationship with CP.

### 4.2. Colocalization Analysis

Figure 3E illustrates the colocalization analysis outcomes of CP and *GZMK*: H0 = 1.44e − 99, H1 = 2.82e − 100, H2 = 0.99, H3 = 0.00, H4 = 0.008. The posterior probability (H4) of shared causal variants between CP and *GZMK* was estimated to be 0.8%. The colocalization analysis results of CP and *KLRG1* are as follows: H0 = 3.23e − 05, H1 = 3.80e − 09, H2 = 0.95, H3 = 5.98e − 05, H4 = 0.05. The posterior probability of 5% was obtained for the shared pathogenic genetic variants between CP and *KLRG1*. The colocalization analysis results of CP and *LYAR* are as follows: H0 = 3.47e − 302, H1 = 7.16e − 307, H2 = 0.99, H3 = 1.52e − 05, H4 = 0.005. The posterior probability of 0.5% was obtained for the shared pathogenic genetic variants between CP and *LYAR*.

### 4.3. Constructing Cell Communication and Pseudotime Analysis

**Figure 4:**
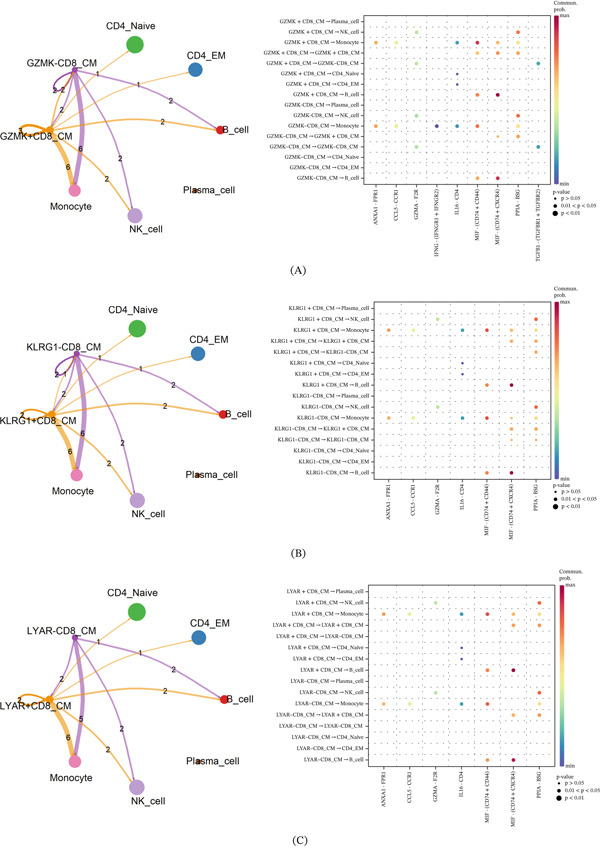
Cell‐cell communication trajectories are as follows: (A) *GZMK +* CD8_CM cell and *GZMK-*CD8_CM; (B) *KLRG1 +* CD8_CM cell and *KLRG1-*CD8_CM; (C) *LYAR +* CD8_CM cell and *LYAR-*CD8_CM.

Through cell communication analysis, Figures 4A validates the interactions between *GZMK* + CD8_CM, *GZMK*‐CD8_CM, and other cell subsets. Figures 4B shows the interactions between*KLRG1* + CD8_CM, *KLRG1*‐CD8_CM, and other cell subsets. Figures 4C shows the interactions between *LYAR* + CD8_CM, *LYAR*‐CD8_CM, and other cell subsets. Regardless of whether *GZMK*, *KLRB1*, and *LYAR* are in high or low expression states, there are differences in the main communication pathways of CD8_CM cells. For example, the main genetic pathways involved in cell communication between *GZMK* + CD8_CM cells and monocyte are *ANXA1-FPR1*, *MIF-(CD74 + CD44),* and *MIF-(CD74 + CXCR4)*; whereas the main communication genetic pathways between *GZMK*‐CD8_CM cells and monocyte are *ANXA1-FPR1* and *MIF-(CD74 + CD44)*. The main genetic pathway involved in cell communication between *KLRG1* + CD8_CM and *KLRG1*‐CD8_CM cells is *PPIA–BSG*; whereas the main communication genetic pathways between *KLRG1*‐CD8_CM and *KLRG1*‐CD8_CM cells are *MIF-(CD74 + CXCR4)* and *PPIA-BSG*. The main genetic pathways involved in cell communication between *LYAR* + CD8_CM cells and Monocyte are *ANXA1-FPR1*, *MIF-(CD74 + CD44)* and *MIF-(CD74 + CXCR4)*; whereas the main communication genetic pathways between *LYAR*‐CD8_CM cells and Monocyte are *ANXA1-FPR1* and *MIF-(CD74 + CD44)*.

**Figure 5:**
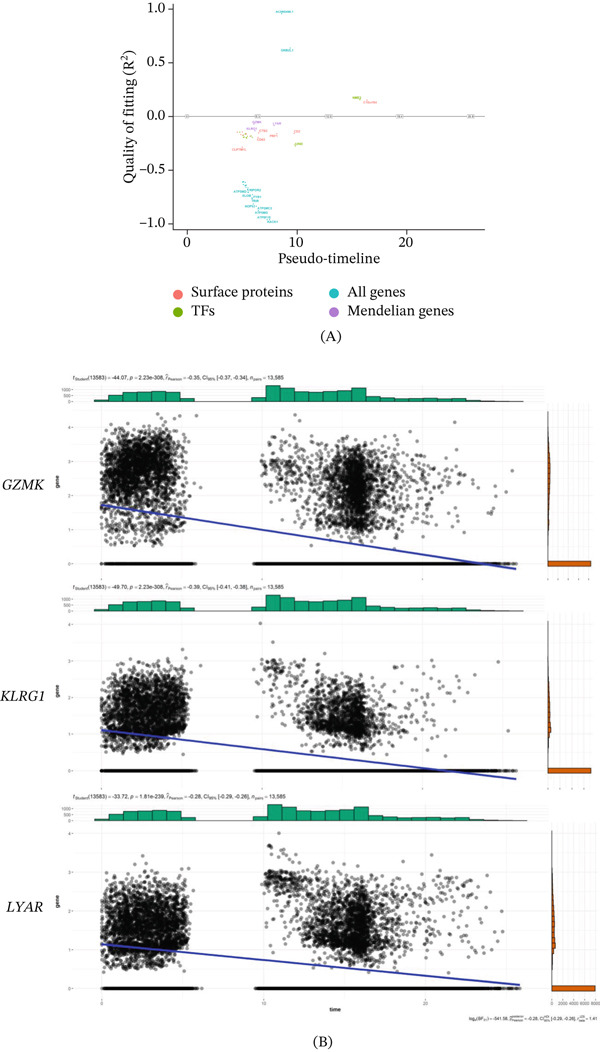
Pseudotime trajectory analysis between chronic periodontitis (CP) and aging. (A) Timeline of differentially expressed genes. (B) Expression pattern of the genes along pseudotime.

Pseudotime trajectory analysis uncovered a distinctive temporal expression pattern of cell cycle‐related genes: *GZMK*, *LYAR, and KLRG1* were significantly expressed in the early stage (0–10) (Figure 5A), suggesting that the above three genes may be involved in cell cycle initiation. As shown in Figures 5B, with the progression of pseudotime, the expressions of *GZMK, KLRG1*, and *LYAR* genes decreased significantly (Pearson correlation coefficients were −0.35, −0.39, and −0.28; *p* values were 2.23e−308, 2.23e−308 and 1.81e−239, respectively), which also suggests that these three genes may be involved in the relatively early stage of the cell trajectory.

### 4.4. Enrichment Metabolism Pathway Analysis of Key Genes

As illustrated in Figure S1, *GZMK, KLRG1,* and *LYAR* are mainly expressed in CD8_CM cells. Distinct sets of predominantly enriched signaling pathways were observed in CD8_CM cells, contingent on the high versus low expression status of these three genes. Consistent with the data presented in Figure S2A, CD8_CM cells with high *GZMK* expression were predominantly enriched in the following pathways: ubiquinone and other terpenoid‐quinone biosynthesis, terpenoid backbone biosynthesis, pantothenate and CoA biosynthesis, other glycan degradation, glycosphingolipid biosynthesis‐lacto and neolacto series, glycosphingolipid biosynthesis‐ganglio series, glycosaminoglycan degradation and glycosaminoglycan biosynthesis‐keratan sulfate. CD8_CM cell with lower *GZMK* expression was mainly enriched in the following pathways: glycosphingolipid biosynthesis‐globo and isoglobo series and glycosphingolipid biosynthesis‐ganglio series. Compared with the *GZMK* subsets, *GZMK +* CD8_CM cells exhibited prominent enrichment in pathways related to energy metabolism, membrane lipid remodeling, and cofactor‐dependent redox regulation. These metabolic features enable sustained effector function, enhanced tissue infiltration, and resistance to oxidative stress, thereby contributing to persistent inflammation, periodontal tissue destruction, and the progression of periodontitis. CD8_CM cell exhibiting elevated *KLRG1* expression showed significant enrichment in the subsequent pathways: ubiquinone and other terpenoid‐quinone biosynthesis, terpenoid backbone biosynthesis, pantothenate and CoA biosynthesis, glycosphingolipid biosynthesis‐ganglio series, glycosaminoglycan degradation, glycosaminoglycan biosynthesis‐keratan sulfate and drug metabolism ‐ other enzymes. CD8_CM cell exhibiting decreased *KLRG1* expression showed primarily enrichment in the subsequent pathways: other glycan degradation, one carbon pool by folate, nicotinate and nicotinamide metabolism, metabolism of xenobiotics by cytochrome P450, glycosphingolipid biosynthesis‐lacto and neolacto series and drug metabolism‐cytochrome P450. *KLRG1*+ CD8_Exhau cells show significantly higher metabolic reprogramming than KLRG1‐ CD8_Exhau cells. Such metabolic reprogramming enables *KLRG1 +* CD8_CM cells to persistently survive in the hypoxic nutrient‐deprived inflammatory microenvironment of periodontitis, while sustaining their immune memory and effector functions. CD8_CM cells with high *LYAR* expression were predominantly enriched in the following pathways: ubiquinone and other terpenoid‐quinone biosynthesis, terpenoid backbone biosynthesis, nicotinate and nicotinamide metabolism, glycosphingolipid biosynthesis‐lacto and neolacto series, glycosphingolipid biosynthesis‐ganglio series, glycosaminoglycan degradation, glycosaminoglycan biosynthesis‐keratan sulfate and drug metabolism‐other enzymes. CD8_CM cell with lower *LYAR* expression was mainly enriched in the following pathways: pantothenate and CoA biosynthesis, other glycan degradation, one carbon pool by folate and drug metabolism‐cytochrome P450.Compared with the *LYAR-* subsets, *LYAR +* CD8_CM cells are significantly enriched in the aforementioned metabolic pathways, which are directly or indirectly involved in the pathological processes of periodontitis. As shown in Figure S2B, although the expression levels of *KLRG1*, *GZMK*, and *LYAR* were increased in the CP group, the differences were not statistically significant.

## 5. Discussion

CP is a common chronic inflammatory disease that not only affects people′s quality of life and oral hygiene but also is closely associated with other systemic diseases [20, 21]. Approximately 10% of the global population is estimated to be affected by this condition, with age being a significant risk factor for increased incidence. To investigate the association between CP and aging, we centered our efforts on elucidating their common mechanisms and immune mechanisms [20, 22]. Firstly, we integrated multiple scRNA‐seq datasets using established analytical approaches. Referring to previous studies [23–26], we prioritized the T cell population for analysis. Compared with HC, the distribution of T cell subsets in both the CP cohort and the aging cohort showed significant changes, among which the substantial decrease in the proportion of CD8_CM cells was particularly notable. Subsequent analyses revealed overlapping DEGs between CP and aging. To further identify the core genes that may play a key role in the pathogenesis of CP, we conducted MR analysis. Finally, we performed metabolic enrichment analysis to explore the interplay between these key genes (*GZMK*, *KLRG1*, and *LYAR*), immune cells, and metabolic pathways in the context of CP and aging. This integrated research approach has revealed the common molecular and immune characteristics between CP and aging, providing insights related to potential therapeutic targets for the precise treatment of CP in the future.

CD8_CM cells, which highly express CCR7 and CD62L, are mainly enriched in secondary lymphoid organs such as the blood, lymph nodes, and spleen, which represent a long‐lived subset of memory cytotoxic T cells characterized by self‐renewal and strong proliferative capacity [27]. Previous study [28] has reported that the proportion of CD8_CM cells in the peripheral blood of patients with CP was higher than that in healthy controls, and periodontal treatment could reduce the percentage of CD8_CM cells. These findings suggest that CD8_CM cells may serve as a peripheral immune marker for disease activity and therapeutic efficacy in CP. In contrast, our study observed a decrease in CD8_CM cells in patients with CP. This discrepancy may reflect the distinct effects of different disease backgrounds and activity levels on the proportion and subset distribution of CD8^+^ memory T cells [29–31]. It is possible that our data captured an active disease state characterized by persistent antigenic stimulation, which drives the differentiation of CD8_CM cells toward effector or exhausted phenotypes, thereby reducing the number of central memory cells. Periodontal treatment may alleviate the antigenic burden and facilitate the reconstitution of memory T cell subsets. CD8_CM cells derived from CP produce only interferon‐*γ* (IFN‐*γ*), which is closely associated with accelerated alveolar bone resorption during the progression of CP [20, 32, 33]. IFN‐*γ* recognized as a key pro‐inflammatory factor in aging can promote cellular senescence [34–36] and senescent cells can drive sterile chronic inflammation [37]. These above suggest that aging may represent a potential risk factor for CP. Although Chiu et al. [38] have reported that the number of CD8_CM cells was significantly elevated with aging, this discrepancy may be explained by the fact that more than 90% of CD8_CM cells in aged mice were virtual memory T cells, whereas the existence of virtual memory T cells in humans remains unclear. In addition, inherent differences between mice and humans may account for the inconsistency, as relevant studies in the human population are still lacking.

*GZMK* belongs to the serine protease family and is predominantly expressed in T lymphocytes. Beyond its intrinsic cytotoxic activity, extracellular *GZMK* has pro‐inflammatory effects [39]. Studies have reported that *GZMK* exhibits the potential to trigger protease‐activated receptor‐1 (PAR‐1) in endothelial cells and fibroblasts, provoking the secretion of inflammatory cytokines including IL‐1, IL‐6, monocyte chemoattractant protein‐1 (MCP‐1), and TNF‐*α* [40], which belong to the critical pro‐inflammatory factors in CP. In addition, *GZMK* exerts a pro‐inflammatory effect via cleavage of extracellular substrates such as LPS, PAR1, and complement proteins. LPS is another extracellular substrate of *GZMK* [41, 42], and is considered as one of the core pathogenic factors driving inflammation and destruction of periodontal tissues. These findings suggest that *GZMK* may trigger the continuous amplification of inflammation in CP. Compared with young individuals, the expression of *GZMK* in CD8+ T cells of the elderly is increased. *GZMK* is capable of aggravating the aging‐associated secretory phenotype of fibroblasts, and in the context of aging, *GZMK* may potentiate the inflammatory response either independently or in synergy with IFN‐*γ* [43]. Our results indicate that *GZMK* is related to CP and aging, and it is necessary to conduct further research on it in the setting of immunosenescence and cancer immunology. A further insight into the molecular mechanisms underlying *GZMK* function could unlock novel therapeutic strategies for the management of CP and age‐related immune dysfunction.

*KLRG1* is an inhibitory lectin‐like Type II transmembrane glycoprotein receptor, and mainly expressed in NK cells and T cell subsets. Its primary functions include delivering costimulatory and coinhibitory signals, thereby regulating the activation and proliferation of immune cells in response to self and foreign antigens, and participating in cell‐mediated immune responses [44–46]. Functionally, as an immune checkpoint receptor, *KLRG1* binds to its ligand cadherin and regulates immune cell proliferation and immune responses via the phosphatidylinositol 3‐kinase/protein kinase B (PI3K/AKT) signaling pathway [47–50]. As is well known, the PI3K/AKT pathway is critically involved in CP. Sustained activation of PI3K/AKT enhances osteoclastogenesis and promotes the survival of inflammatory cells in periodontal tissues, thereby exacerbating tissue destruction and bone loss [51–54]. These findings suggest that *KLRG1* may participate in alveolar bone resorption in CP. Furthermore, the expression of *KLRG1* is positively correlated with aging. Senescent CD8^+^ T cells exhibit elevated expression of senescence markers, and *KLRG1* is among the most highly expressed markers in terminally differentiated T cells [47, 55]. T cells expressing *KLRG1* also show a 2.5–6.25‐fold higher capacity to secrete inflammatory cytokines such as IFN‐*γ*, thus promoting a pro‐inflammatory state [56]. Further studies exploring the relationship between *KLRG1* and CP as well as aging from an immunological perspective may provide novel insights into the age‐related changes in CP.

*LYAR* is a nucleolar and nuclear protein harboring a zinc‐finger DNA‐binding motif and functions as a transcription factor with a specific DNA‐binding domain. It plays critical roles in cell growth regulation, ribosome biogenesis, gene expression control, and tumorigenesis [57, 58]. As a negative regulator of the innate immune response, *LYAR* has been reported to negatively regulate IFN‐*β*‐mediated immune responses by suppressing the DNA‐binding activity of interferon regulatory factor 3 (IRF3). Additionally, *LYAR* inhibits nuclear factor kappa (NF‐*κ*B)‐driven expression of pro‐inflammatory cytokines such as IL‐6, IL‐8, IL‐1*β*, and TNF‐*α* [59]. Notably, IFN‐*β* is involved in immune suppression in CP [60], and NF‐*κ*B represents a core pro‐inflammatory signaling pathway in CP [61, 62]. Moreover, experimental evidence indicates that *LYAR* represses the transcription of oxidative stress‐related genes through binding to their promoters [63]. Oxidative stress serves as a key player in the pathophysiology of aging and represents one of the molecular mechanisms underlying age‐related disorders [64, 65]. Further studies are therefore warranted to characterize the role and potential mechanisms of *LYAR* in the pathophysiological processes of CP and aging from an immunological standpoint.

This study adopted a variety of innovative analysis strategies. Firstly, integrated bulk RNA with single‐cell transcriptome datasets, these results pinpointed codysregulated genes (such as *KLRG1*, *GZMK*, *LYAR*) as key targets for subsequent research. Secondly, through time‐series analysis of the progressive stages of CP and the aging stage, we showed stage‐dependent gene expression characteristics and the phenomenon of impairing intercellular crosstalk and associated such patterns with immunosenescence and chronic inflammation. This time‐course analysis also screened out prognostic‐related genes with time dependence, which may have therapeutic potential. Finally, through cell communication modeling, we revealed the regulatory network concentrated on genes *GZMK*, *KLRG1*, and *LYAR*, including their impact on key signaling cascades, providing a systematic perspective for identifying combined therapeutic strategies. Although these merits are acknowledged, the study′s limitations should also be considered. First, aging is a complex and multifactorial process, and the interaction between immune senescence and periodontitis may involve additional cell types and signaling pathways not fully captured in this study. Second, the colocalization analysis results for the three genes showed that their H4 values all approached 0. The findings indicated that although these three genes may have certain phenotypic associations, their genetic regulatory mechanisms are independent of each other. This discovery suggests that when analyzing the occurrence and development mechanisms of related diseases, these genes may function through distinct biological pathways rather than sharing a unified genetic regulatory module. Therefore, future mechanistic studies need to further focus on their respective unique signaling pathways and molecular interaction networks to precisely elucidate their specific pathogenic mechanisms. Third, we acknowledge that our study mainly focuses on CP, which may limit the generalizability of our findings to other forms of periodontitis. Finally, the findings of this study are primarily based on computational analyses and require further experimental validation to confirm the biological functions and mechanisms of the identified key genes.

## 6. Conclusion

Comparative analysis of differential gene expression between CP and aging cohorts revealed a panel of coregulatory genes (*GZMK*, *KLRG1*, and *LYAR*) and delineated their putative roles in CP pathogenesis and aging progression. This study facilitates an in‐depth comprehension of the association between aging and CP and lays a foundation for precise intervention.

## Author Contributions

**Fengzhen Lei:** writing – review and editing, writing – original draft, formal analysis. **Rui Shi:** writing – original draft, formal analysis. **Qiuli Chen:** writing – review and editing, writing – original draft. **Zelong Zeng:** visualization, validation, formal analysis. **Chen Sun:** writing – original draft, visualization, validation, supervision, conceptualization. **Mei He:** writing – review and editing, writing – original draft, visualization, validation, supervision, conceptualization.

## Funding

This work was supported by the Scientific Research Projects of Medical and Health Institutions of Longhua District, Shenzhen (No. 2026047).

## Conflicts of Interest

The authors declare no conflicts of interest.

## Supporting information


**Supporting Information** Additional supporting information can be found online in the Supporting Information section. Supporting Information. Figure S1: The UMAP of the gene expression level (*GZMK*, *KLRG1* and *LYAR*). Supporting Information. Figure S2: The bulk‐RNA analysis in chronic periodontitis (CP). (A) The major enrichment metabolic pathways of CD8_CM cell expressing and not expressing *GZMK*, *KLRG1* and *LYAR* and other T cell types. (B) The heat map of expression levels.

## Data Availability

The data that support the findings of this study are available on request from the corresponding author. The data are not publicly available due to privacy or ethical restrictions.
