# Supplementary material for: Identifying Shared Key Genes and Cellular Characteristics Between Chronic Periodontitis and Aging Using Integrative Single‐Cell and Mendelian Randomization Analyses
Source: Biomed Res Int. 2026 Jun 17;2026:5156800. doi: 10.1155/bmri/5156800 (PMC13273837; doi:10.1155/bmri/5156800)
Supplement: Supplementary file 1 — Supporting Information Additional supporting information can be found online in the Supporting Information section. Supporting Information. Figure S1: The UMAP of the gene expression level (GZMK, KLRG1 and LYAR). Supporting Information. Figure S2: The bulk‐RNA analysis in chronic periodontitis (CP). (A) The major enrichment metabolic pathways of CD8_CM cell expressing and not expressing GZMK, KLRG1 and LYAR and other T cell types. (B) The heat map of expression levels. [file BMRI-2026-5156800-s001.pptx]

## Slide 1
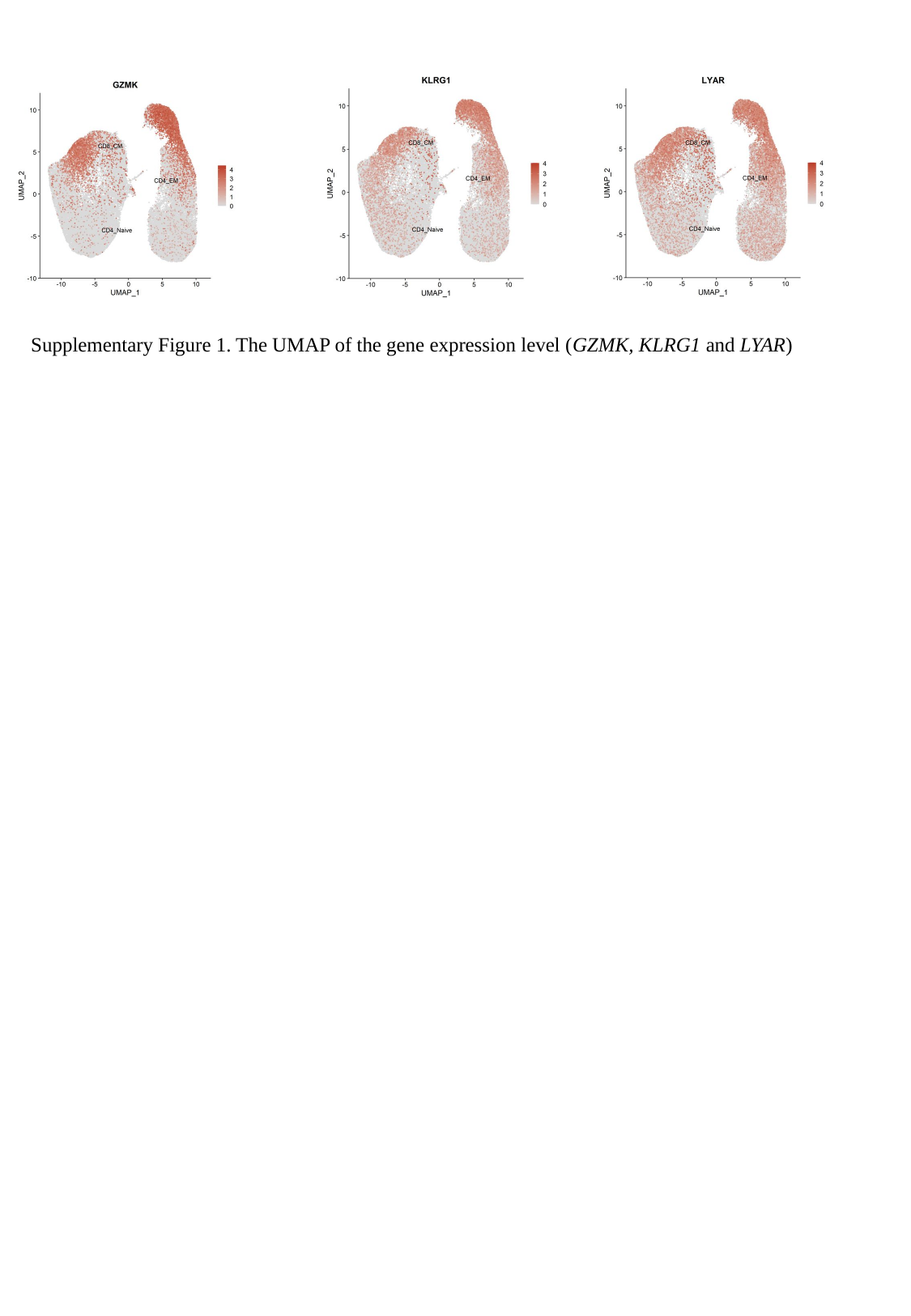

Supplementary Figure 1. The UMAP of the gene expression level (GZMK, KLRG1 and LYAR)

## Slide 2
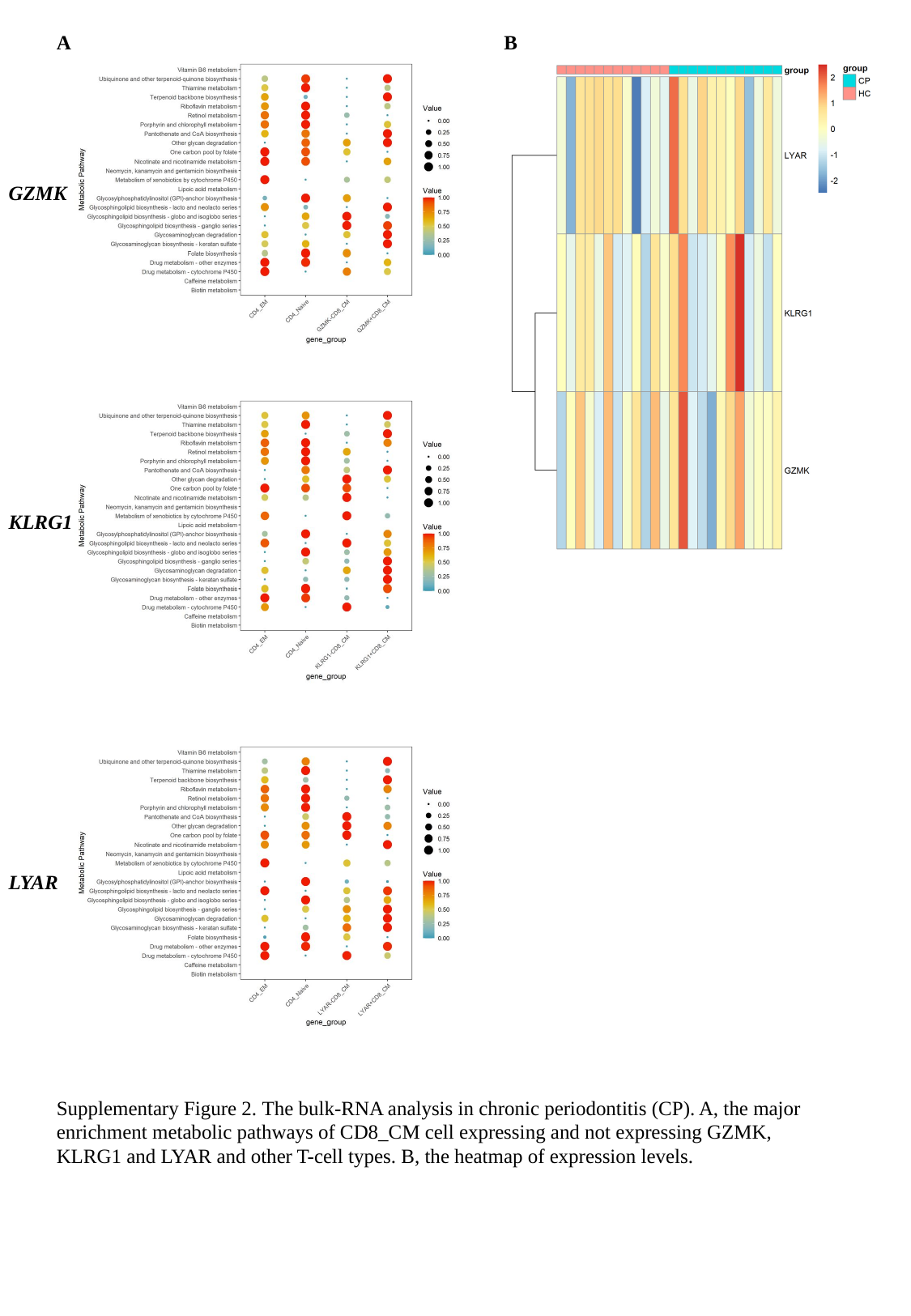

A
B
GZMK
KLRG1
LYAR
Supplementary Figure 2. The bulk-RNA analysis in chronic periodontitis (CP). A, the major enrichment metabolic pathways of CD8_CM cell expressing and not expressing GZMK, KLRG1 and LYAR and other T-cell types. B, the heatmap of expression levels.
